# *Clostridioides difficile* surveillance: 9-year comparison between automated surveillance and conventional surveillance in acute care hospitals

**DOI:** 10.1017/ash.2025.5

**Published:** 2025-02-24

**Authors:** Jean Stanciu, Patrick Dolcé, Charles Frenette, Marie-Claude Roy, Lina Kouider, Yves Longtin

**Affiliations:** 1 Medical Microbiology and Infectious Diseases, Centre Intégré de Santé et de Services Sociaux du Bas-Saint-Laurent, Rimouski, QC, Canada; 2 Medical Microbiology and Infectious Diseases, McGill University Health Centre, Montreal, QC, Canada; 3 Medical Microbiology and Infectious Diseases, CHU de Québec-Université Laval, Quebec City, QC, Canada; 4 Medical Microbiology and Infectious Diseases, Infection Prevention and Control Unit, Jewish General Hospital, Montreal, QC, Canada

## Abstract

**Objective:**

To develop and validate an automated surveillance system for healthcare-associated *Clostridioides difficile* infections (HA-CDI).

**Design:**

Multicenter cohort study.

**Setting:**

16 acute care hospitals.

**Patients:**

Patients admitted to participating hospitals between 2013 and 2022.

**Methods:**

An automated surveillance system was developed with retrospective extraction from admission/discharge/transfer and laboratory databases and compared with conventional surveillance based on clinical definitions collected prospectively by infection control professionals. Comparison of HA-CDI incidence rates calculated by automated vs conventional surveillances were performed with χ^2^, incidence rate ratios, and linear regression. A subset of discordant cases was further investigated by reviewing medical records.

**Results:**

Overall, conventional surveillance reported 3,211 cases of HA-CDI for an incidence rate of 4.94 per 10,000 patient-days. Automated surveillance detected 4,708 cases, for an incidence rate of 7.24 per 10,000 patient-days (incidence rate ratio, 1.47; 95% CI, 1.40–1.53). Full concordance between both surveillance methods was observed in 62% of cases, while 34% of cases were detected only by automated surveillance, and 4% were detected by conventional surveillance only. Between 2013 and 2022, an identical declining trend in HA-CDI incidence rates of –0.54 cases per 10,000 patient-days was observed with both surveillance methods. A subset of 49 cases detected only by automated surveillance were reviewed; the main reasons for discrepancy were delayed testing (39%), colonization (24%), misclassifications (14%), and interinstitutional transfers (12%).

**Conclusions:**

HA-CDI incidence rates calculated by automated surveillance were higher than those of conventional surveillance, but the overestimation was consistent over time, suggesting that a correction factor could improve precision.

## Introduction

Healthcare-associated infections represent a major but preventable complication that can increase patients’ length of hospitalization, morbidity, and mortality.^1^ *Clostridioides difficile* infections (CDI) remain one of the most prevalent healthcare-associated infection.^2^ To better understand its epidemiology, detect outbreaks, and improve its control, surveillance and reporting of CDI incidence rates have been implemented in numerous jurisdictions.^3,4^ This conventional surveillance is mainly conducted through manual chart review by qualified health professionals.^4^ To increase inter-institutional comparability, recommendations regarding optimal surveillance methods have been published and provide guidance regarding definitions, denominators, and reporting of infection rates.^5,6^ The Institut National de Santé Publique du Québec in the Canadian province of Quebec (population 9 million), implemented a mandatory CDI surveillance program in 2004 following the onset of the NAP1/B1/027 epidemic.^7^ As of August 2023, all 95 acute care facilities admitting >1,000 patients per year have the obligation to participate. This program relies on infection control professionals (ICPs) to assess and report cases of CDI based on standardized case definitions, using a combination of clinical and laboratory criteria, and to classify them into categories such as healthcare-associated CDI (HA-CDI) and community-associated CDI.^7^ Despite being the gold standard, this conventional surveillance method has some drawbacks and is laborious, costly, and time-consuming.^8^ The surveillance definitions also contain some subjective elements (such as the assessment of other causes that could explain the diarrhea) and relies on professionals’ clinical interpretation which makes it susceptible to errors and biases and complicates interinstitutional comparisons.^9–12^ Automated surveillance using computerized algorithms, which could estimate CDI incidence rates using information readily available in healthcare electronic databases without the need for human input and interpretation, could help solve some of these issues by providing an efficient and uniform surveillance method that may reduce the ICPs’ workload.^9,13^ In the United States, the National Healthcare Safety Network of the Centers for Disease Control and Prevention offers 2 options for reporting CDI: an automated electronic laboratory-identified algorithm and a conventional infection surveillance reporting by ICPs.^14^ The current study aimed to develop and validate an automated surveillance system for the detection of HA-CDI in multiple Quebec hospitals.

## Methods

### Setting

We performed a retrospective cohort study of patients admitted between April 1, 2013, and March 31, 2022, in 16 acute care hospitals (10 academic and 6 community) representing 25% of all HA-CDI cases in Quebec in 2022.

### Definitions

For conventional surveillance purposes, CDI is defined as either (1) diarrhea (i.e. ≥3 unformed or liquid stools within 24 hours and symptoms lasting ≥24 hours without any other known etiology) combined with a positive assay for toxigenic *C. difficile* from a stool sample; or (2) visualization of pseudomembranes by colonoscopy; or (3) a histopathologic diagnosis.

A CDI event is categorized as HA-CDI if symptoms appeared ≥3 calendar days after admission and up to 4 weeks after discharge. Cases in which the patient developed symptoms within the first 3 calendar days of admission, and those with no history of hospitalization within the previous 4 weeks, are considered community-associated. Recurrent CDI is defined as a relapse of symptoms < 8 weeks after the end of the previous treatment; these cases are excluded from the surveillance data. Incidence rates are calculated as the number of cases per 10,000 patients-days and are reported per 4-week period. HA–CDI incidence rates per hospital in the province of Quebec are publicly available.^7^ Data from neonatal intensive care units, nurseries, psychiatric wards, long-term care, and ambulatory care are excluded from the surveillance program.

### Automated surveillance

An automated surveillance system was developed using data extracted from Nosokos (Nosotech, Rimouski, Canada), an infection control software already in place in the participating institutions. Data extraction included information for *C. difficile* testing, sampling date, patient location, and dates of admission and discharge. All dates included precision to minutes level. Information regarding bowel habits and diarrhea, colonoscopy reports, or histopathology reports were not available and thus not integrated.

Positive *C. difficile* tests included toxin detection by nucleic acid amplification testing (NAAT) for toxin B gene or enzyme immunoassay (EIA) that detects both toxin A and/or B antigen (ToxAB).^7^ Positive assays for glutamate dehydrogenase enzyme had to be confirmed with a second confirmatory assay (NAAT or ToxAB EIA) to be considered positive.

For automated surveillance, positive toxin tests are considered to represent CDI. A CDI case is categorized as HA-CDI if the sample was collected ≥3 calendar days after admission and up to 4 weeks after discharge. All other cases were considered as outpatient cases or infections acquired in another facility.^7^

**Figure 1.**
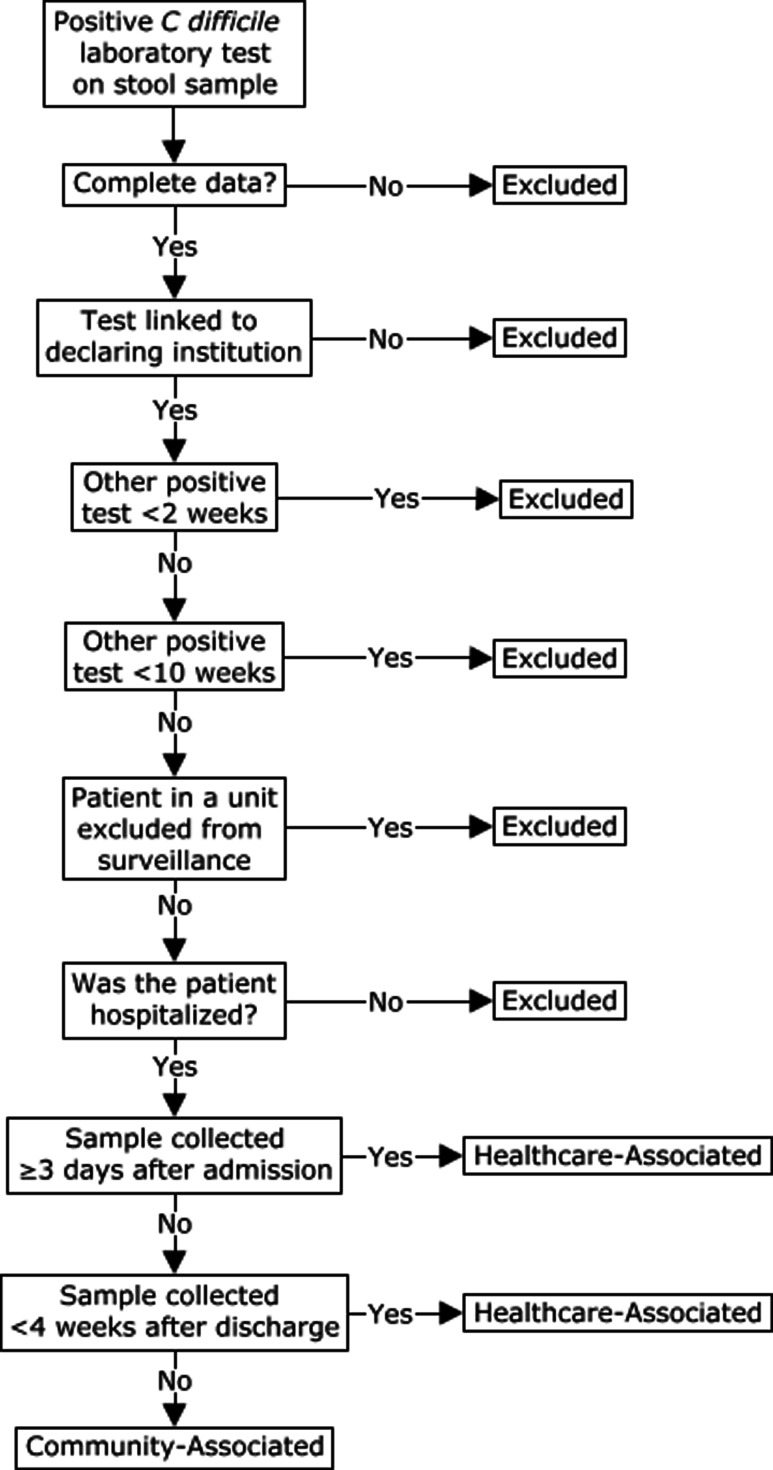
The automated surveillance algorithm for categorization of positive *C. difficile* tests consists of one starting point: a recognized positive *C. difficile* laboratory test. Next are exclusions: for tests that are not recognized, incomplete data, tests not linked to the declaring institution, duplicates (other positive test <2 weeks), recurrences (other positive test <10 weeks), patient in a unit excluded from surveillance, patient that was not hospitalized. If a sample was collected ≥3 calendar days after admission of <4 weeks after discharge, the case was considered healthcare-associated. Remaining tests were considered community-associated.

Automated surveillance was also designed to exclude certain cases: duplicate cases (defined as a second positive *C. difficile* test for the same patient in the same hospital within a 2-week period), recurrent cases (defined as a second positive *C. difficile* test for the same patient in the same hospital within a 10-week period), cases from nonparticipating facilities and non-hospitalized patients, and cases with incomplete data (Figure 1). The denominators, patient-days, used to calculate automated surveillance incidence rates were identical to those from conventional surveillance.

### Analysis

Standard descriptive analyses were performed to describe the number of tests carried out during the study period, the types of assays and the proportion of positive tests. Aggregated data were analyzed for the entire study period to estimate the global HA-CDI incidence rate per 10,000 patient-days for automated surveillance and conventional surveillance by combining data from all 16 institutions. These rates were compared by χ^2^ and incidence rate ratios (IRR) with 95% confidence intervals (CI). We analyzed the capacity of automated surveillance to detect secular trends in HA-CDI incidence by comparing the trends (slopes) in the aggregated incidence between 2013 and 2022 reported by both methods using ANOVA.

At the level of individual institutions, we investigated the capacity of automated surveillance to predict institutional HA-CDI incidence rates reported by conventional surveillance by calculating the overall incidence rates for each hospital using each surveillance method. Each institutional automated surveillance incidence rate was compared with conventional surveillance using Poisson regression. We used a 95% CI on the global institutional rate ratio to detect hospitals whose automated surveillance incidence rate departs more importantly from their conventional surveillance rates than others. To investigate the impact of automated surveillance on each institution’s HA-CDI incidence relative to others, we ranked each hospital from the lowest to the highest incidence based on automated and conventional surveillance methods.

We examined the degree of global concordance between each surveillance method in the attribution of individual HA-CDI cases. CDI cases that were deemed as a HA-CDI case by both methods were considered concordant, whereas those that were considered as HA-CDI in only one of the two methods were considered discordant. The sensitivity, specificity, positive predictive value, and negative predictive value were also calculated in comparison with conventional surveillance as gold standard. A subset of discordant HA-CDI cases was investigated by reviewing the patients’ electronic records to determine the causes of discrepancy.

### Correction factor

Considering that some variables that are essential to assess potential cases of CDI in conventional surveillance are not electronically available (for example, the use of laxatives and the number of bowel movements per day), we expected that automated surveillance would overestimate the number of HA-CDI cases and incidence rates. We explored the effect of applying a correction factor derived from a linear regression on automated surveillance vs conventional surveillance yearly HA-CDI infection rates aggregated by hospital groups. The quality of fit of the linear model on the data was evaluated with the coefficient of determination (R^2^). We then compared the corrected automated surveillance HA-CDI annual infections rates with conventional surveillance infection rates.

The institutional research ethics committees for each participating institution approved the retrospective extraction of data for analysis with a waiver of individual patient consent.

## Results

During the study period that included 6.49 million patient-days of surveillance, a total of 140,125 *C. difficile* laboratory tests were performed in the participating institutions, of which 15,145 were positive, for an overall positivity rate of 11%. Of these positive tests, 50.8% were NAAT for toxin B gene and 49.2% were ToxAB EIA. During the study period, 3,211 cases of HA-CDI were reported by conventional surveillance. By comparison, automated surveillance detected 4,708 possible HA-CDI cases. Automated surveillance detected 2,185 non-nosocomial CDI cases while conventional surveillance reported 2,413 such cases. By contrast, 8,293 cases were excluded by automated surveillance while 9,562 were excluded by conventional surveillance.

The overall HA-CDI incidence rate reported by conventional surveillance was of 4.94 per 10,000 patient-days. Automated surveillance had an incidence of 7.24 per 10,000 patient-days, for an IRR (HA-CDI detected by automated surveillance divided by HA-CDI detected by conventional surveillance) of 1.47 (95% CI, 1.40–1.53; *P* < .001).

### Comparison of HA-CDI incidence rates over time

**Figure 2.**
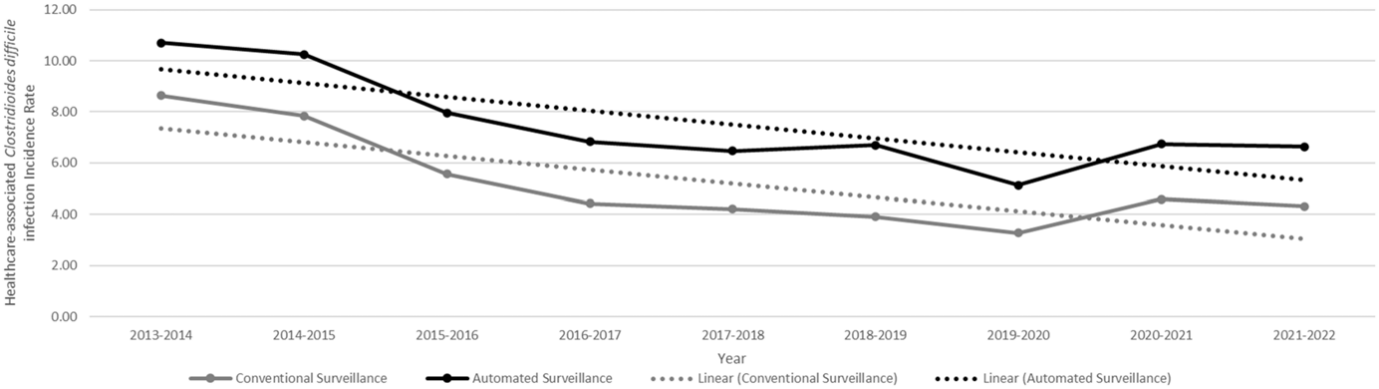
Linear graph illustrating the annual aggregated healthcare-associated *Clostridioides difficile* infection (HA-CDI) incidence rates calculated by automated vs conventional surveillance methods among 16 hospitals in Quebec, Canada over a 9-year period. Linear trendlines to detect secular trends in HA-CDI incidence are also shown with dotted lines for both methods.

The HA-CDI incidence followed a similar downward trend in each surveillance method (Figure 2). Over the course of the study period (9 years), the aggregated HA-CDI rates reported by conventional surveillance decreased at a rate of –0.54 cases per 10,000 patient-days (95% CI, –0.90 to –0.17, *P* = .01). Similarly, HA-CDI incidence calculated by automated surveillance also decreased by –0.54 cases per 10,000 patient-days (95% CI, –0.90 to –0.19; *P* = .01). There was no difference between the decreasing trend detected by both methods (*P* = .98 by ANOVA).

### Interinstitutional comparison of HA-CDI incidence rates

**Figure 3.**
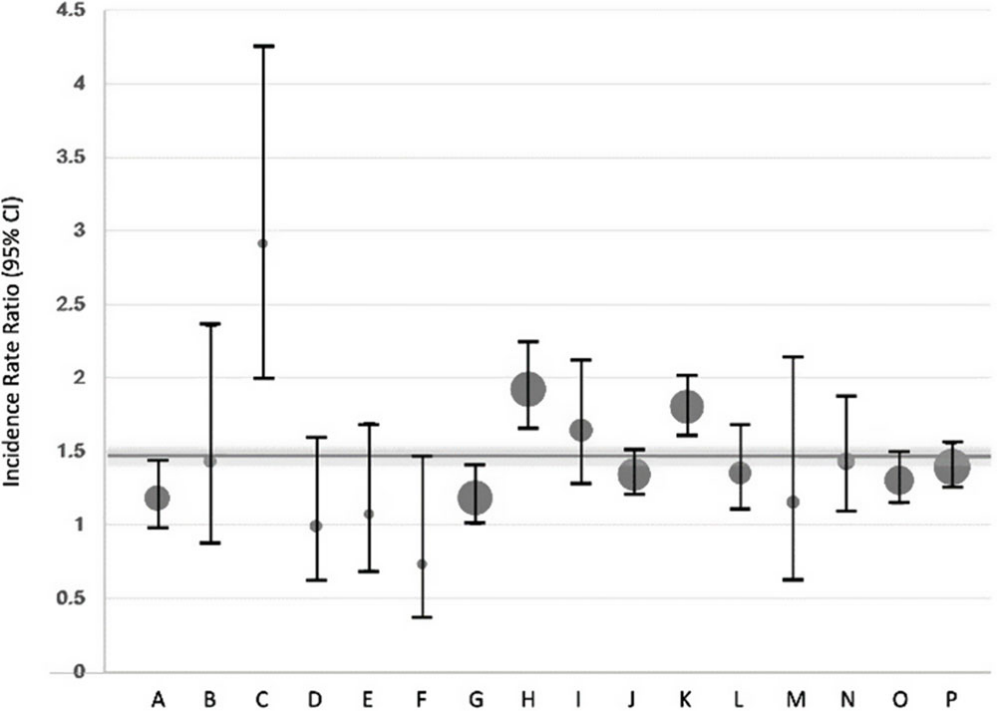
Incidence rate ratio (IRR) and 95% confidence intervals (CI) for healthcare-associated *Clostridioides difficile* infections (HA-CDI) incidence rate calculated by automated vs conventional surveillance. The overall IRR and 95% CI is illustrated by the horizontal bars. The sample size of each institution is illustrated by the size of the dot.

**Figure 4.**
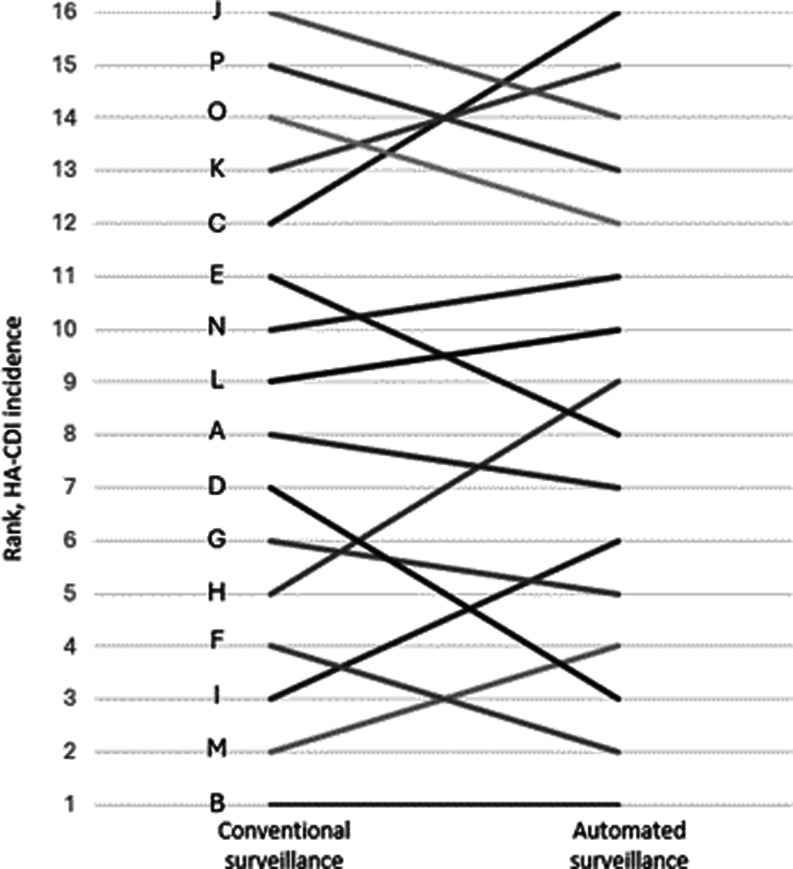
Sankay diagram illustrating the change in overall ranking of participating institutions when healthcare-associated *Clostridioides difficile* infections (HA-CDI) incidence rates are computed by automated versus conventional surveillances.

The IRR were also calculated for each hospital (Figure 3). Although the range of IRR varied between 0.74 and 2.92, there was a general trend for automated surveillance to detect a higher HA-CDI incidence in most institutions. The IRR was significantly greater than 1 in 10/16 (63%) institutions, whereas only a single institution had an IRR <1. Three institutions had an IRR that was significantly higher than their peers’. The greater discrepancy among these institutions was associated with a change in ranking relative to their colleagues, from 12th to 16th position, 5th to 9th, and from 13th to 15th, respectively (Figure 4). Overall, only a single hospital occupied the same ranking in both surveillance strategies (1st in both systems), while the greatest movement in ranking was a position change of 4 (from 12th to 16th position).

### Global concordance of HA-CDI cases and performance of automated surveillance

Looking at the global concordance of HA-CDI cases identified by either automated or conventional surveillance, 62% of HA-CDI cases were concordant, 34% were detected by automated surveillance only, and 4% were detected by conventional surveillance only. Thus, automated surveillance overestimates the number of cases, but infrequently misses a HA-CDI case detected by conventional surveillance. Using conventional surveillance as gold standard, automated surveillance showed a 93.9% sensitivity, 84.4% specificity, 64.1% positive predictive valuem and 97.9% negative predictive value.

### Causes of discrepancy

We reviewed a sample of 49 discrepant cases detected only by automated surveillance, in a single center to gain insight on the potential cause(s) of divergence between the two methods. The main reasons for discrepancy were (1) a delay between the onset of symptoms and the collection of a stool sample for *C. difficile* testing (39%), (2) patients colonized by *C. difficile* rather than being infected (24%), (3) misclassification of HA-CDI (14%), (4) interinstitutional transfers (12%), and (5) miscellaneous reasons such as manual errors, recurrent cases, or cases diagnosed in other institutions (10%). An example of delayed testing would be a patient with CDI symptoms appearing on the second day of hospitalization, but for which a stool sample was collected on Day 3 and tested positive for *C. difficile*, and was thus classified as nosocomial by automated surveillance.

### Correction factor

**Figure 5.**
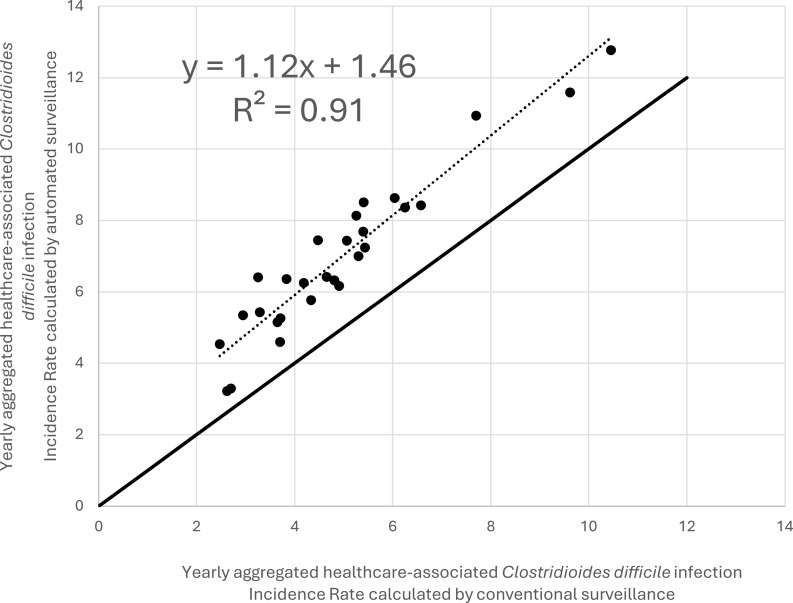
Scatter plot illustrating yearly healthcare-associated *Clostridioides difficile* infections (HA-CDI) incidence rates calculated by automated vs conventional surveillance methods for each group of hospitals among 16 hospitals in Quebec, Canada over a 9-year period. Linear regression yielded a linear trendline equation of y = 1.12x + 1.46, where y represents HA-CDI incidence rates calculated by automated surveillance and x represents HA-CDI incidence rates calculated by conventional surveillance. Coefficient of determination (R^2^) = 0.91.

**Figure 6.**
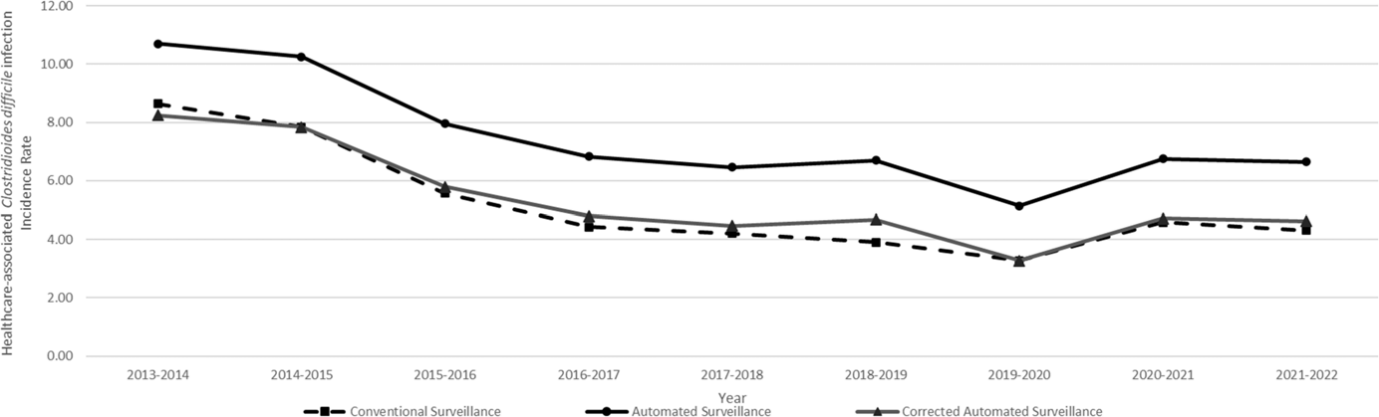
Linear graph illustrating the annual aggregated healthcare-associated *Clostridioides difficile* infection (HA-CDI) incidence rates calculated by conventional surveillance, automated surveillance, and automated surveillance adjusted with the correction factor among 16 hospitals in Quebec, Canada over a 9-year period.

Linear regression analysis of yearly data aggregated by groups of hospitals showed a strong positive association between HA-CDI rates estimated by automated surveillance vs conventional surveillance, with a coefficient of determination (R^2^) of 0.91 (Figure 5). This linear regression yielded a linear trendline equation of y = 1.12x + 1.46, where y represents HA-CDI incidence rates calculated by automated surveillance and x represents HA-CDI incidence rates calculated by conventional surveillance. Using this formula, we hypothesized that the application of a correction factor could improve the automated surveillance’s capacity to estimate HA-CDI incidence rates as measured by conventional surveillance. This correction factor would consist in subtracting 1.46 from the HA-CDI incidence rate calculated by automated surveillance and dividing this result by 1.12. This correction of automated surveillance would provide a more accurate estimate of conventional surveillance. (Figure 6).

## Discussion

CDI surveillance is widely conducted across jurisdictions. Most current programs rely on trained auditors, a method that is time-consuming and laborious, as it relies on humans to detect and assess potential cases of CDI. The possibility to conduct automated surveillance has drawn interest of researchers worldwide.^15^ Although we found good concordance between the results of automated and conventional surveillances, automated surveillance overestimated the number of HA-CDI cases by 47%. These results are in line with previous studies that found that automated HA-CDI algorithms tend to overestimate the number of HA-CDI cases, by proportions ranging from 29% to 73%.^9,13,16–18^ A retrospective study conducted in 2019 in another Canadian province, Alberta, found that automated surveillance overestimated by 43% when compared to conventional surveillance.^17^

We found that the causes of the overestimation of automated surveillance were not unique to our algorithm and have been reported previously in other papers.^9,13,17–21^ These included delay between the onset of symptoms and the collection of a sample for testing, testing of patients who did not fulfill the case definition for CDI (for example due to the presence of <24h of diarrhea), testing in other institutions, manual errors, and recurrent cases.^9,13,17–21^ Delayed testing and patients not fulfilling a CDI definition were the main causes of discrepancies that were found in our population. In fact, assessment of symptomatology is not currently included in most automated surveillance methodologies, which makes it presently challenging to differentiate between infection and colonization. However, it must be stressed that these limitations are likely to be addressed in the future by adding additional databases as hospitals gradually move from written medical charts to electronic ones. Hence, it is plausible that automated surveillance will become more precise in the future.

The comparison of HA-CDI incidence rates detected by automated surveillance between NAAT and ToxAB EIA testing were not possible, since many hospitals used both techniques over the years. Many of them shifted from EIA to NAAT, and the reported HA-CDI rates to Public Health did not specify the testing methodology.

As clinical information is not readily available electronically, HA-CDI incidence rates calculated with automated surveillance are unlikely to match a manual method. However, our study identified that the overestimation is relatively stable over time, and that a simple mathematical correction factor can improve the algorithm’s precision and provide a relatively accurate estimate of the actual HA-CDI incidence rates, especially as the sample size increases.

Large-scale implementation of automated surveillance for HA-CDI could help strengthen the current surveillance program by achieving two main goals: to observe the evolution of infections through time and to compare infection rates between hospitals more uniformly. In fact, automated surveillance provides clear, objective, and identical criteria to each hospital whereas conventional surveillance may differ due to the professionals’ personal judgment and may be affected by cognitive biases.^11,12^ Therefore, implementing automated surveillance may make comparisons between different hospitals easier and more reliable.

Most CDI surveillance programs do not include in-depth quality assessment of the data reported by participating institutions. Our study identified inter-hospital variation in the automated to conventional surveillance HA-CDI incidence rate ratios. Even though the causes of this discrepancy remain unclear at the moment, this observation raises the possibility that automated surveillance algorithms could be used to detect institutions that underreport or overreport hospital-acquired infections. This information could become valuable to monitor the quality of data reported by ICPs.^22^ It is important to stress that even the current “gold standard” contains some level of subjectivity. Hence, the variability between the automated and conventional surveillances does not necessarily reflect flaws in the algorithm but could also reflect, in part, variations in patient populations or in the local application of the surveillance method and definitions. It is difficult to estimate which method is closer to reality.^23^

Our study has strengths. By spanning nearly a decade and enrolling 16 hospitals, it identified that automated surveillance could detect secular trends in incidence that are nearly identical to those seen by conventional surveillance. It also identifies strategies that take advantage of automated surveillance for quality monitoring, and determined that correction factors could be potentially applied to improve the precision of automated surveillance. It also has limitations. Confirmation of the usefulness of correction factors should be performed on an external validation cohort. This study did not analyze the real-world impact of the implementation of the algorithm, such as the impact on ICPs’ workload, the acceptability by IT teams, ICPs and the Ministry of Health, and the feasibility of a large-scale implementation. Still, we believe our results are valuable to help understand the potential future uses of this emerging technology.

In summary, this multicenter study identified that automated surveillance could provide useful to improve various aspects of *C. difficile* infection surveillance programs. Further studies are required to gain further insight on this promising technology.

## Data preparation

Nosotech inc. provided assistance with data gathering and preparation.
